# Diverse Roles and Therapeutic Potentials of Circular RNAs in Urological Cancers

**DOI:** 10.3389/fmolb.2021.761698

**Published:** 2021-11-19

**Authors:** Song Wang, Yufan Ying, Xueyou Ma, Weiyu Wang, Xiao Wang, Liping Xie

**Affiliations:** Department of Urology, The First Affiliated Hospital, School of Medicine, Zhejiang University, Hangzhou, China

**Keywords:** circular RNAs, urological cancers, biomarker, therapy targets, mechanism

## Abstract

Circular RNAs (circRNAs) are a novel class of noncoding RNAs, which are mainly formed as a loop structure at the exons caused by noncanonical splicing; they are much more stable than linear transcripts; recent reports have suggested that the dysregulation of circRNAs is associated with the occurrence and development of diseases, especially various human malignancies. Emerging evidence demonstrated that a large number of circRNAs play a vital role in a series of biological processes such as tumor cell proliferation, migration, drug resistance, and immune escape. Additionally, circRNAs were also reported to be potential prognostic and diagnostic biomarkers in cancers. In this work, we systematically summarize the biogenesis and characteristics of circRNAs, paying special attention to potential mechanisms and clinical applications of circRNAs in urological cancers, which may help develop potential therapy targets for urological cancers in the future.

## Introduction

Recently, plentiful researches have delineated sophisticated regulatory networks formed by diverse RNA species, including protein-coding messenger RNAs (mRNAs) and noncoding RNAs represented by circular RNAs (circRNAs) and microRNAs (Tay et al., 2011; Luigi et al., 2018; Xu et al., 2019; Zhao Q. et al., 2020; Vitiello et al., 2020). Emerging evidence showed that noncoding RNAs make up the majority of the total transcriptome, whereas only 2% of the human transcriptome are protein-coding mRNAs (Boley et al., 2014). With a deeper understanding of the transcriptional landscape, the noncoding RNAs have shown their significances in the pathogenesis of diverse human diseases, especially in cancers (Slack and Chinnaiyan, 2019). We previously elucidated that microRNAs (miRNAs) are involved in the occurrence and development of urological cancers such as renal cell carcinoma (RCC), bladder cancer (BC), and prostate cancer (PCa) (Li J. et al., 2017; Wang S. et al., 2017; Zhu et al., 2020), whereas the circRNAs in urological tumors remain poorly understood.

As rising stars in noncoding RNAs, circRNAs have attracted more and more attention from researchers all over the world. However, they were traditionally considered as nonfunctional “genomic junks” generated by abnormal splicing events and until specific-expressed circRNA in testicular tissue was found to have potential functions (Capel et al., 1993; Wilusz and Sharp, 2013). Recently, novel analysis such as the RNase R-treated and non-polyadenylated transcriptomes analysis have determined thousands of circRNAs in a great diversity of eukaryotes, including humans (Jeck et al., 2013; Vicens and Westhof, 2014; Zhang et al., 2014), and discovered them to have species-specific, tissue-specific, and time-specific expression patterns (Chen, 2016). Meanwhile, plentiful researches elucidated that the circRNAs are extensively involved in cancer cell growth and innate immunity, and their dysregulation is closely correlated with disease occurrence and progression (Dube et al., 2019; Huang et al., 2019; Vo et al., 2019; Wilusz, 2019), especially the initiation and development of human cancers (Guarnerio et al., 2019; Rajappa et al., 2020), including urological cancers (Chen S. et al., 2019; Lu et al., 2019; Li J. et al., 2020).

CircRNAs are mainly derived from nonconventional back-splicing, where the downstream site is ligated to the forward acceptor site (Vicens and Westhof, 2014). Remarkably, circRNAs were reported to express independently of homologous linear isoforms, and they can accumulate to high expression levels due to their complete closed-loop fabric, which endows them more tolerant to deterioration by exonucleases. It has been proposed that naturally expressed circRNAs not only can serve as endogenous miRNA sponges, or competing endogenous RNAs (ceRNAs), but also can act as protein antagonists or translation templates to regulate physiological and pathological activities (Chen, 2020). Understanding this novel regulatory network may yield insights into intricate gene regulatory systems in human development and disease progression. In our comprehensive work, we summarized the latest findings of circRNAs in biogenesis and characteristics, with a focus on their potential mechanisms and clinical applications in urological cancers, which may help us to develop potential therapy targets for urological cancers in the future.

## Circular RNAs

### Origination, Classification, and Regulation of Circular RNAs

**FIGURE 1:**
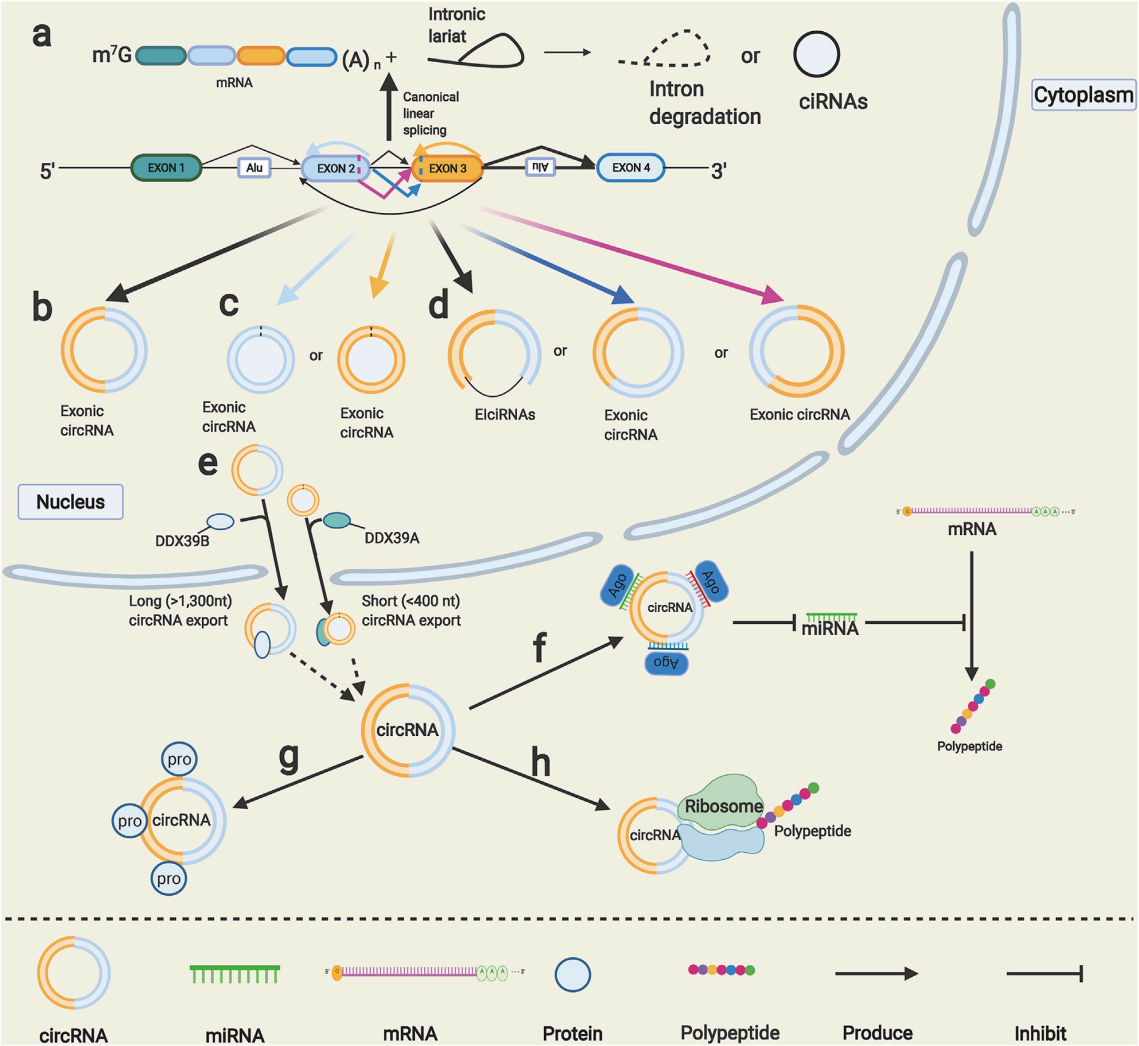
Formation and functions of circRNAs. **(A)** Canonical linear splicing can produce normal linear mRNA **(left)** and intronic lariat **(right)**, intronic lariat rapidly degraded after splicing or escape from debranching step then generate circular intronic RNAs (ciRNAs) **(right)**. **(B)** Back-splicing of exons produces circRNAs. **(C)** Alternative back-splice yields different single-exon circRNAs. **(D)** Alternative splice that happened within circRNAs produces three basic types of circRNAs. **(E)** Output of circRNAs from nucleus to cytoplasm requires unique proteins mediation. **(F)** CircRNAs can function as miRNA sponge to indirectly regulate miRNA downstream target genes. **(G)** CircRNAs can directly interact with proteins to regulate their functions. **(H)** CircRNAs can translate unique peptides.

Most eukaryotic genes contain one or more introns among multiple exons, and canonical linear splicing mechanisms usually remove the introns and ligate the exons together to yield mature mRNAs (Figure 1A). Although the competitions between linear splicing and noncanonical splicing have always existed in human transcriptome, as the conventional linear splicing events slow down, the circular RNAs will be produced by an alternative nonconventional splicing event called back-splicing, in which a backward donor site is covalently linked to a forward acceptor site (Tay et al., 2014) (Figure 1B). Simultaneously, intron lariats resected during canonical splicing can escape debranching and retain the circular form in a few cases, which are dubbed as circular intronic RNAs (ciRNAs) (Zhang et al., 2013) (Figure 1A). Notably, although the odds are slight, a given gene locus that includes multiple exons can yield diverse circRNAs by a special form of back-splicing called alternative back-splicing (Gao et al., 2016); through this mechanism, each exon splices and combines its own set of 5′ donors and 3′ acceptors, thus producing circRNAs with back-splice sites from a single exon (Figure 1C). In addition, splicing sites within the circRNAs will also generate three novel circRNAs, including intron retention, alternative 5′ and 3’ splicing circRNAs, unlike conventional back-splicing (Zhang et al., 2016) (Figure 1D). When it comes to the classification of circRNAs, we generally focus on the components of them, and the circRNAs can be easily divided into three types: 1) exonic circRNAs, they are mainly generated in the nucleus and then exported to the cytoplasm; they can function as sponges for diverse miRNAs and proteins (Chen et al., 2015). 2) ciRNAs, are almost nonfunctional and distributed in the nucleus (Li X. et al., 2021). 3) exon–intron circRNAs (ElciRNAs), found to interact with small nuclear ribonucleoproteins and bind to RNA polymerase II to regulate gene transcription (Li Z. et al., 2015). Although the nucleus is the back-splicing events factory, most functional circRNAs are distributed in the cytoplasm. Recently, some researchers have proposed that the abundance of circRNAs was determined by the steady-state regulatory system composed of circRNA generation, nuclear export, and degradation (Conn et al., 2015; Chen, 2020). On the one hand, intronic complementary sequences have been proved to facilitate the generation of circRNAs (Zhang et al., 2014), and many RNA-binding proteins can regulate the production of circRNAs through binding to intronic complementary sequences. These include positive regulatory protein QKI, HNRNPL, and negative regulatory protein DHX9, ADAR1 (Conn et al., 2015; Rybak-Wolf et al., 2015; Aktaş et al., 2017; Fei et al., 2017). On the other hand, the circRNA nuclear export mechanisms remained unclear until a novel published study reported that circRNAs could transport from the nucleus to the cytoplasm depending on their length (Huang et al., 2018) (Figure 1E). CircRNAs can stably exist thus amass high expression levels because of their loop structures, which help them be tolerant to exonucleases leaded RNA degradation; several studies showed that the miRNA endonucleases might also be involved in circRNA degradation mechanisms (Hansen et al., 2011; Park et al., 2019).

### Biological Properties of Circular RNAs

CircRNAs possess some unique biological characteristics that are different from other RNA: 1) circRNAs show a wide variety of diversity, more than a million circRNAs have been reported in the circAtlas database (Wu et al., 2020), and over a million cancer-specific circRNAs in the CSCD2 database (Feng et al., 2021). 2) High stability, circRNAs possess a longer half-life in comparison with linear RNA due to their loop structure (Enuka et al., 2016). 3) Highly conserved, a recent study showed that the sequences of some circRNAs were highly conserved among multiple species (Jeck et al., 2013). 4) Expression specificity, different tissues were reported to have a variable abundance of circRNAs (Salzman et al., 2013). For example, cirs-7 was detected as highly expressed in neural tissues while decreased in other tissues (Memczak et al., 2013).

### Functions of Circular RNAs

CircRNAs can serve as miRNA sponges. As early as 2007, researchers found an intriguing phenomenon called “target mimicry,” in which a noncoding RNA can sequester miR-399 and release its downstream targets (Franco-Zorrilla et al., 2007). In 2011, Leonardo et al. unified the hypothesis about how gene coding mRNAs and noncoding RNAs coregulate and affect each other by using MREs as “communication media.” In that research, the term “competing endogenous RNA” (ceRNA) was coined to describe this new layer of a regulatory network across the transcriptome (Salmena et al., 2011). Henceforth, more and more circRNAs were identified to function as ceRNAs, also known as miRNA sponges that bind corresponding target miRNAs and indirectly regulate the miRNAs target genes (Figure 1F). CircRNAs can also directly interact with proteins to cause their degradation or enhance their functions (Wang X. et al., 2021) (Figure 1G). In addition, some circRNAs were reported to have internal ribosome entry site elements and can be the templates for translation (Gao X. et al., 2021; Jiang T. et al., 2021; Wu et al., 2021) (Figure 1H). CircRNAs have been widely reported to be involved in regulating transcription factor activity, cell proliferation, epithelial-to-mesenchymal transition (EMT), and stem cells through targeting specific signaling pathways and genes. Chen et al. (2021) demonstrated that cia-MAF drives liver tumor progression *via* transcription factor MAFF; CircZFR was found to promote BC cell proliferation by targeting transcription factor ZEB2 (Zhang W.-Y. et al., 2019). CircPTCH1 and circCSNK1G3 were reported to promote the EMT process in RCC (Liu H. et al., 2020; Li W. et al., 2021). CDR1as and hsa_circ_0003222 were found to regulate stemness and development of lung cancer (Zhao Y. et al., 2020) (Li C. et al., 2021). Additionally, a novel circular RNA, named circSETD3, was reported to inhibit stem cell properties, cell growth, and EMT in BC (Tian et al., 2021). These studies showed that circRNAs are implicated in regulating cell proliferation, EMT, and stem cell properties by multiple mechanisms.

### Circular RNAs and Exosomes

Exosomes are a kind of vesicles that are generated and secreted by almost all cell types; they are packed with a series of materials, including circRNAs, and act on the recipient cells, thus exerting biological functions (Zhang Y. et al., 2019). Emerging evidence demonstrated that exosome-derived circRNAs play vital roles in the progression of cancer (Reese and Dhayat, 2021). Gao L. et al. (2021) reported that the exosome-derived circCOG2 drives colorectal cancer progression by activating transforming growth factor-beta (TGF-β) signaling. Zang et al. (2021) found that exosomes that transferred circ_0000337 lead to cisplatin resistance of esophageal cancer. Exosome circ_0044516 was reported to drive PCa progression by targeting miR-29a-3p (Li T. et al., 2020). In contrast, Jiang Z. et al. (2021) reported that exosomal circEPB41L2 could inhibit colorectal cancer development by regulating the PTEN/AKT pathway. Several recent studies have shown that exosomes contain highly elevated expression of circRNAs due to their high protective properties, making exo-circRNAs promising biomarkers and targets for therapy (Li Y. et al., 2015; Shi et al., 2020).

## Roles of Circular RNAs in Human Urological Cancers

Dysregulation of circular RNAs was reported to be implicated in the occurrence and development of urological cancers (Chen S. et al., 2019; Lu et al., 2019; Li J. et al., 2020); they were found to have extensively participated in the growth, apoptosis, invasion, and migration of urological cancers.

### Renal Cell Carcinoma

RCC is one of the most common malignant tumors of the urinary system, accounts for 5% of male adult malignant tumors and 3% of female adult malignant tumors around the world (Siegel et al., 2020). Recently, some circRNAs have been reported to regulate RCC through affecting PI3K/AKT/mTOR signaling, which is involved in tumor proliferation, migration, and apoptosis, including activator circHIPK3 and inhibitor hsa-circ-0072309 (Chen T. et al., 2019). Circ-ZNF609 was reported to act as a competitive miR-138-5p sponge and suppress its expression, then release FOXP4 and promote the progression of RCC (Xiong et al., 2019), whereas cRAPGEF5, derived from the RAPGEF5 gene, was downregulated in RCC and the sponged miR-27a-3p released, resulting in decreased expression of TXNIP and blocking the progression and migration of RCC (Chen Q. et al., 2020). Notably, microarray analysis identified that the upregulation of circRNA hsa_circ_001895 (Chen Z. et al., 2020) and circPCNXL2 (Zhou et al., 2018) could, respectively, sponge the miR-296-5p and miR-153, led to the liberty of transcription factor SOX12, ZEB2, and thus promote RCC proliferation.

**FIGURE 2:**
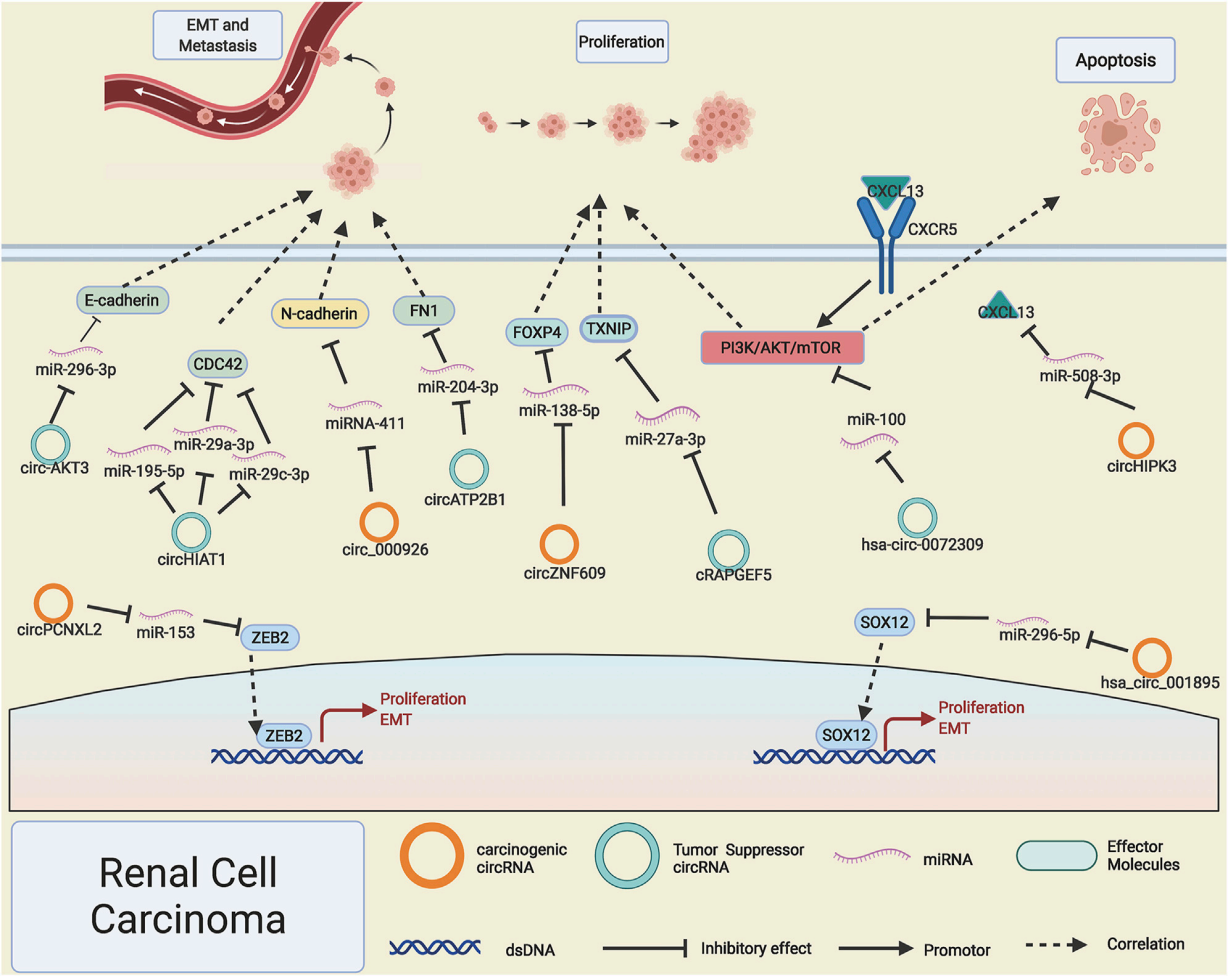
Possible mechanisms of circRNAs in renal cell carcinoma. Upregulated circPCNXL2, circZNF609, hsa_circ_001895, circ_000926, and circHIPK3 (orange rings) and downregulated hsa-circ-0072309, cRAPGEF5, circ-AKT3, circATP2B1, and circHIAT1 (cyan rings) regulate progression of renal cell carcinoma by altering specific gene expression or tumor-related signaling pathways.

Invasion and migration are particularly important characteristics in the occurrence and progression of malignant tumors; our previous research has reported that miRNA could affect the invasion and migration of RCC (Zhu et al., 2020); as the upstream regulatory molecule of miRNA, circRNAs have been observed to participate in the process of EMT and metastasis of RCC. Xue et al. (2019) reported that the lowly expressed circ-AKT3 in RCC leads to the freedom of its adsorbed miR-296-3p, which results in the reduction of EMT negative marker's expression and significantly increase the RCC tumor EMT process. However, highly expressed circ_000926 promotes the EMT process by boosting the expression of N-cadherin (Zhang D. et al., 2019). Studies have shown that the weak adsorption capacity of circHIAT1 causes the elevated expression of miR-195-5p/miR-29a-3p/mi-R29c-3p, which indirectly leads to decreased CDC42 expression and inhibits the invasion and migration of RCC (Wang K. et al., 2017). Intriguingly, Han et al. (2018) found that circATP2B1 can stabilize miR-204-3p, which is not in accord with the conventional ceRNA theory; they reported that highly expressed ERβ suppress the expression of circATP2B1 and then led to the reduction of miR-204-3p, which caused the increased expression of EMT positively related marker FN1 and thus promoted RCC cell invasion (Figure 2).

### Bladder Cancer

BC is a malignant tumor that is particularly prone to recurrence, which ranked the top 10 cancers all over the world, with extremely high mortality and morbidity (Barone et al., 2015). However, studies on early diagnosis of BC and specific markers are still lacking, and current treatments for BC are also unsatisfactory; it is important to further explore precise strategies for gene regulatory networks in BC. As a rising star in cancer, circRNAs also play pivotal roles in the progression of BC. CircTFRC was reported to be significantly upregulated in 57 cases of BC tissues compared with adjacent normal tissues, and with the increase of tumor stages and grades, its expression level enhanced significantly; miR-107 was proved to be sponged by highly expressed circTFRC, which led to the downstream target gene TFRC release and drive the progression of BC, coincidentally; the target gene TFRC was the ancestral gene of circTFRC (Su et al., 2019). PI3K/AKT signaling pathway is a common and important transduction pathway for diversified cancer cell proliferation and metastasis (Zhang et al., 2017). Yang C. et al. (2019) applied RNA interference technology to silence the expression of circUVRAG. Downregulation of circUVRAG led to the upregulation of miRNA-223 and suppression of fibroblast growth factor receptor 2, which is an important activator of the PI3K/AKT signaling pathway. CircRNA circSLC8A1 was confirmed to be lowly expressed in nearly 100 pairs of BC tissues compared with adjacent normal tissues, which led to the discharge of downstream sponged miR-494, miR-130b, and the depression of PTEN. PTEN has been widely considered as a tumor suppressor gene and negatively regulates the PI3K/AKT pathway (Lu et al., 2019).

EMT and angiogenesis processes are important hallmarks of BC progression (Yang et al., 2020). CircPRMT5 can drive the EMT process of BC by sponging miR-30c and liberating the transcription factor snail (Chen X. et al., 2018). CircHIPK3 contains two key miR-558 response elements due to its expression level being too low to sponge enough miR-558, resulting in lowly expressed HPSE inhibiting invasion and migration of BC cells (Li Y. et al., 2017). Interestingly, circHIPK3 has been reported to play a role in driving tumor progression both in PCa and RCC (Liu F. et al., 2020; Han et al., 2020). Highly expressed circMYLK and circ0001429 in BC can enhance VEGFA gene expression by, respectively, sponging miR-29a and miR-205-5p, resulting in enhanced tumor angiogenesis process (Zhong et al., 2017; Cao W. et al., 2019).

**FIGURE 3:**
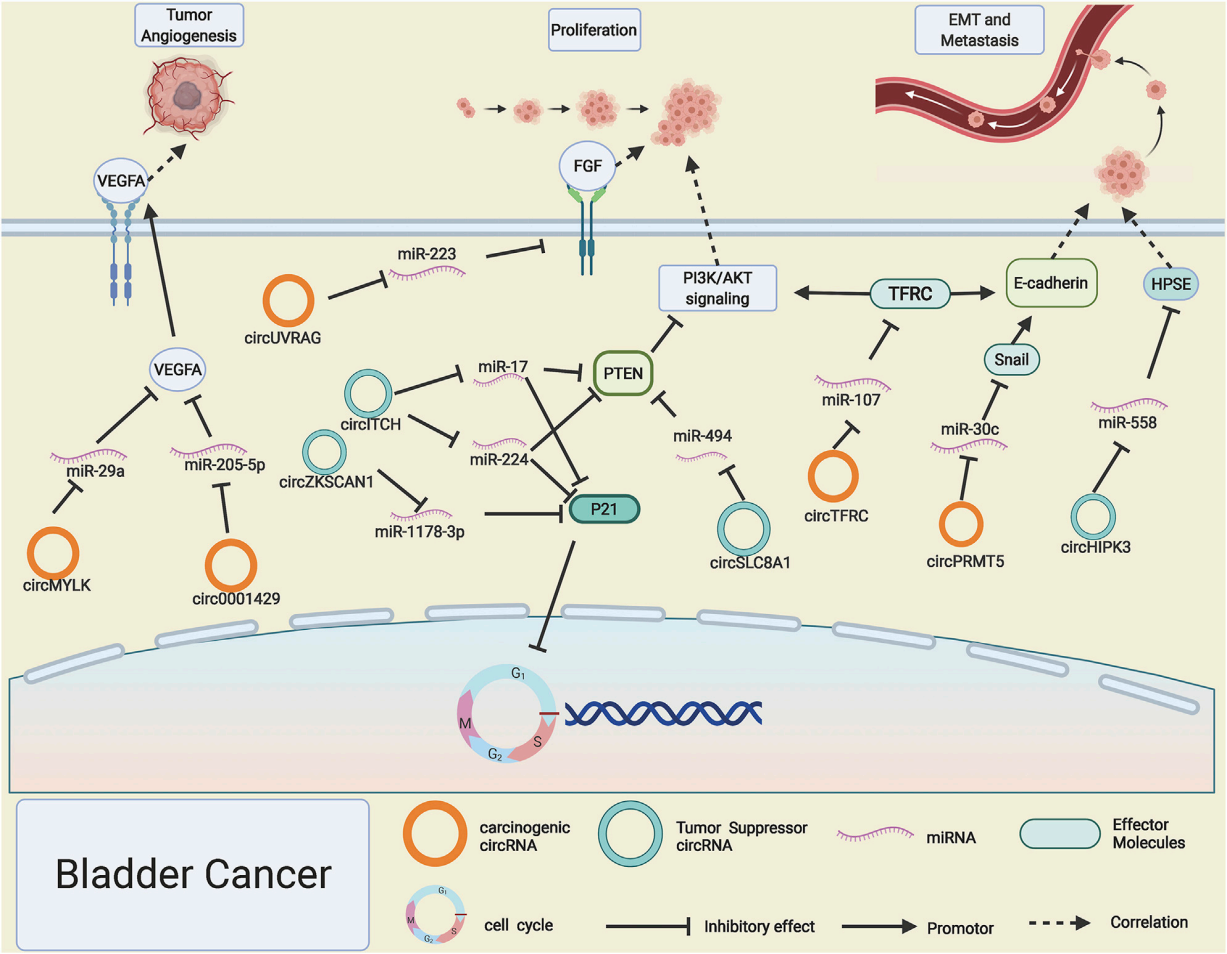
Possible mechanisms of circRNAs in bladder cancer. Upregulated circMYLK, circ0001429, circUVRAG, circTFRC, and circPRMT5 (orange rings) and downregulated circHIPK3, circSLC8A1, circ-ITCH, and circ-ZKSCAN1 (cyan rings) regulate progression of bladder cancer by altering specific gene expression or tumor-related signaling pathways.

The ability to proliferate nearly infinity is a crucial trait of cancer cells, and the disturbance of the cell cycle acts as a vital role in the excessive proliferation of cancer cells (Sanli et al., 2017). With the expansion of ceRNA network research, a wealth of circRNAs have been found to regulate BC cell proliferation by influencing critical molecules of the cell cycle regulation. Circ0058063 functions as a competitive miR-145-5p sponge to restrain its freedom in the cytoplasm, which further leads to the release of cell cycle positively related gene CDK6 (Sun et al., 2019), thus increasing BC cell growth. In contrast, circNR3C1 is dramatically reduced in BC, and it impairs the growth of BC tumor cells by restraining miR-27a-3p to inhibit its interactions with CCND1 and thus suppress cyclin D1 expression (Zheng et al., 2019). P21 (CDKN1A) and P27 (CDKN1B) are known as negative regulators that could induce cell cycle arrest at the G1/S phase and block tumor cell growth. Xie et al. (2018) demonstrated that the BCRC-3 is downregulated in BC, which is capable of acting as a miR-182-5p sponge to influence the expression of p27, thus blocking the growth of BC cells. CircZKSCAN1 and circITCH boost the expression of P21, thus playing a role in BC inhibition by function as sponging for miR-1178-3p and miR-224/miR-17. Simultaneously, circITCH can also enhance PTEN expression, an inhibitor of PI3K/AKT signaling, thus delaying the BC progression (Yang et al., 2018; Bi et al., 2019) (Figure 3).

### Prostate Cancer

PCa is one of the most frequent malignancies with the highest newly diagnosed cases in men and ranked the second cancer-related deaths worldwide (Fizazi et al., 2020). Although dramatic progress has been made in the diagnosis and treatment of PCa, there is still a low overall survival rate among PCa patients. It is extremely vital to discover molecular diagnostic biomarkers and novel treatment targets related to the occurrence and progression of PCa. Like other cancers, circZNF609 is also highly expressed in PCa; it drives the progression of PCa by functioning as a competitive miR-186-5p sponge and giving rise to YAP1 upregulation and activating the AMPK signaling pathway (Jin et al., 2019). CircFOXO3 and circFMN2 can, respectively, sponge miR-29a-3p and miR-1238 and then release SLC25A15 and transcription factor LHX2, which drive the progression of PCa (Kong et al., 2020; Shan et al., 2020). However, when the expression level of circITCH in PCa is declined, the corresponding sponged miR-17-5p is released, which lead to the downregulated gene expression level of HOXB13 and significantly inhibit the development of PCa (Wang et al., 2019); intriguingly, HOXB13 was proved to be a pivotal upstream regulator of AR-V7 and participate in the development of castration-resistant prostate cancer (CRPC) (Chen Z. et al., 2018; Navarro and Goldstein, 2018). Like RCC (Han et al., 2020), circHIPK3 is also found to be overexpressed in PCa; it can promote the proliferation and invasion potential of PCa cells through binding with miRNA-338-3p and enhancing the gene expression level of ADAM17, thus prompting the progression of PCa (Liu F. et al., 2020). However, lowly expressed circ_0007494 in PCa was reported to act as a molecular sponge for miR-616 and thus accelerate the expression of PTEN, which will block the PI3K/AKT signaling pathway, thus preventing tumor progression (Zhang et al., 2020).

**FIGURE 4:**
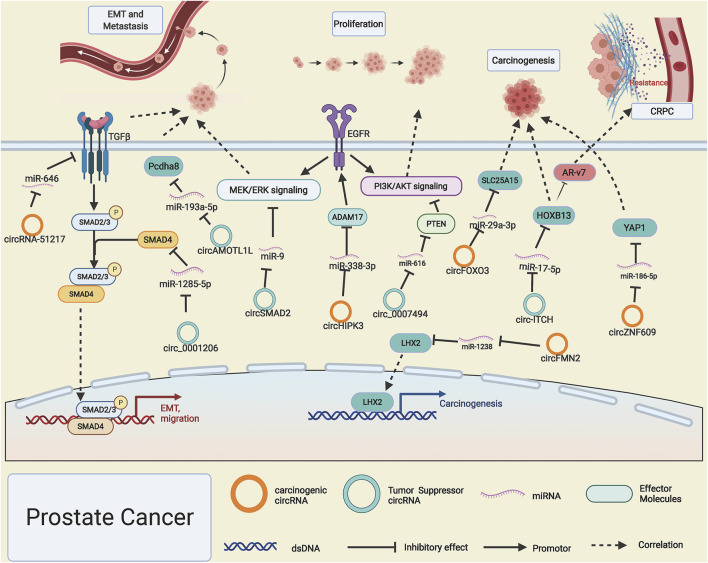
Possible mechanisms of circRNAs in prostate cancer. Upregulated circRNA-51217, circZNF609, circHIPK3, circFMN2, and circFOXO3 (orange rings) and downregulated circ-ITCH, hsa_circ_0001206, circSMAD2, circAMOTL1L, and circ_0007494 (cyan rings) regulate progression of prostate cancer by altering specific gene expression or tumor-related signaling pathways.

The EMT process involves a series of changes such as critical gene expression patterns and cell proliferation, apoptosis, and migration. EMT-related genes' expression is regulated by intricate networks included but not limited to growth factors, transcription factors signaling pathways (Dongre and Weinberg, 2019). Xu et al. (2020) reported that R-2-hydroxyglutarate could enhance the expression level of circRNA-51217, which acts as a ceRNA to absorb miRNA-646 and leads to the upregulation of TGF-β1, thus activating TGF-β1/p-Smad2/3 signaling to enhance the abilities of PCa cell invasion. Furthermore, the androgen receptor (AR) could reverse this process by suppressing the expression of circ RNA-51217. In contrast, circ0001206 was found to function as a cancer suppressor by sponging miR-1285-5p and subsequently boosting SMAD4 expression (Song et al., 2019), which is a well-known cancer suppressor gene in PCa. CircSMAD2, originated from SMAD2 gene transcripts, impedes the EMT process of PCa by sponging miR-9 and abating the phosphorylation of STAT3, MEK, and ERK (Han et al., 2019). Yang Z. et al. (2019) declared that p53/RBM25 signaling is involved in the biogenesis of circAMOTL1L, and downregulated circAMOTL1L leads to the upregulation of dissociative miR-193a-5p, thus inhibiting the expression of Pcdha8 and EMT process of PCa (Figure 4).

## Clinical Applications of Circular RNA in Urological Cancers

In the past few years, a good deal of dysregulated circRNAs have been reported in urinary tumors, including RCC, BC, and PCa, and the expression level of these annotated circRNAs is closely linked to cancer stages, grades, and prognosis, indicating that circRNA can be used for diagnosis and treatment, some circRNAs that might act as biomarkers, and therapeutic targets are listed in Table 1.

**TABLE 1:** CircRNAs act as biomarkers and therapeutic targets in urological cancers.

Cancer type	circRNA	Dysregulation	Sample sources	Function	Clinicopathological association	References
Renal cell carcinoma	hsa_circ_001895	upregulated	tissue, cell lines	prognostic marker and novel ccRCC therapy	TNM stage, OS	Chen et al. (2020b)
	circHIPK3	upregulated	tissue, cell lines	prognostic biomarker and target for the molecular treatment of ccRCC	Tumor stage, grade and size, Overall survival, ROC curve indicates AUC = 0.95322	Han et al. (2020)
	cRAPGEF5	down-regulated	tissue, cell lines	prognostic biomarker and novel therapeutic target for RCC patients	Tumor size, TNM stage, Metastasis, OS, RFS	Chen et al. (2020a)
	circHIAT1	down-regulated	tissue, cell lines	biomarker and potential new therapy	Tumor stage, Metastasis, OS	Wang et al. (2017a)
Bladder cancer	hsa_circ_0137439	upregulated	urine, tissue, cell lines	independent prognostic predicator	Tumor stage, Tumor Grade, Lymph node status, NMIBC/MIBC, OS, RFS	Song et al. (2020)
	circPRMT5	upregulated	urine, plasma, tissue, cell lines	prognostic biomarker and exploitable therapeutic target	Clinical tumor stage, Tumor metastasis, Survival	Chen et al. (2018a)
	cTFRC	upregulated	tissue, cell lines	potential marker of BC diagnosis or progression	Tumor stage, Grade, Lymphatic invasion, Survival	Su et al. (2019)
	circRNA-MYLK	upregulated	tissue, cell lines	promising target for BC diagnosis and therapy	Tumor stage, Overall survival	Zhong et al. (2017)
	circ-BPTF	upregulated	tissue, cell lines	potential biomarker and therapeutic target	Tumor grade, NMIBC/MIBC, Recurrence, Overall survival	Bi et al. (2018)
	circACVR2A	down-regulated	tissue, cell lines	prognostic biomarker and therapeutic target	Clinicopathological characteristics, Overall survival	Dong et al. (2019)
	circ-ZKSCAN1	down-regulated	tissue, cell lines	promising marker and therapeutic target	Tumor grader, Stage, Lymph nodes status, Recurrence, OS, DFS	Bi et al. (2019)
Prostate cancer	circZMIZ1	upregulated	plasma, cell lines	novel biomarker and a treatment target for prostate cancer	correlated with the expression of AR and AR-V7	Jiang et al. (2020)
	circAR3	upregulated	plasma, tissue, cell lines	PCa biomarker	PCa Gleason scores, lymph node metastasis	Luo et al. (2019)
	circFOXO3	down-regulated	tissue, cell lines	potential prognostic and therapeutic approaches for prostate cancer	Tumor grade, chemoresistance to docetaxel	Shen et al. (2020)
	circRNA17	down-regulated	tissue, cell lines	a better therapy for Enzalutamide resistance of PCa	PCa Gleason score, Enzalutamide resistance	Wu et al. (2019)

RCC, renal cell carcinoma. ccRCC, clear cell renal cell carcinoma. BC, bladder cancer. PCa, prostate cancer. OS, overall survival. RFS, recurrence-free survival. DFS, disease-free survival. ROC, receiver operating characteristic curve. AUC, area under the curve. NMIBC, nonmuscle-invasive bladder cancer. MIBC, muscle-invasive bladder cancer. AR, androgen receptor. AR-V7, androgen receptor splice variant 7.

### Circular RNAs Act as Diagnostic and Prognostic Biomarkers in Urological Cancers

With the development of minimally invasive surgical techniques such as robotic surgeries, more and more surgical indications for urinary tumors have been expanded. Owing to a lack of credible and effective ways to early diagnosis, many patients with urinary tumors are still diagnosed with advanced cancer stages and miss the best chance for surgery, which underscores the urgent need for new biomarker detection in urinary cancers. Recently, the potential role of circRNAs as novel biomarkers has been increasingly recognized. Actually, due to their biological features such as high stability and specificity of tissue expression, circRNAs have been found to stably exist in human saliva, urine, and plasma (Wang S. et al., 2021); they are more likely to be ideal diagnostic and prognostic biomarkers. Song et al. (2020) applied microarray to identify differentially expressed circRNAs from 10 cases of BC urine samples and 10 normal urine samples. Subsequently, they discovered that hsa_circ_0137439 is remarkably overexpressed in BC patients' urine samples compared with normal people, which also had been validated in 116 BC tissues and 30 normal tissues. Urinary hsa_circ_0137439 can distinguish not only BC and normal people but also muscle-invasive bladder cancer and nonmuscle invasive bladder cancer; in addition, the expression of has_circ_0137439 is closely associated with recurrence-free survival and overall survival of BC patients. At the mechanism level, hsa_circ_0137439 could function as a miR-142-5p sponge to decrease its expression and thus lead to upregulation of MTDH and promote BC progression, so they proposed that urinary hsa_circ_0137439 could be a new diagnostic and prognostic biomarker and treatment target in BC. Interestingly, Chen et al. (2018) found that circPRMT5 is significantly enriched in serum and urinary exosomes from nearly 100 BC patients samples compared with corresponding normal people, and highly expressed circPRMT5 is in close contact with BC lymph node metastasis and advanced cancer progression. In addition, upregulated cTFRC, circ-BPTF, and downregulated circ-ZKSCAN1 were also identified as promising diagnostic and prognostic biomarkers in BC (Bi et al., 2018; Bi et al., 2019; Su et al., 2019). Cao S. et al. (2019) comprehensively analyzed exome-RNA-seq from 47 cases of metastatic CRPC samples, and RNase R treated RNA-seq of patient-derived xenografts and cell models, then they validated 13 circRNAs derived from the AR in PCa, due to the high detectability of these AR-originated circRNAs in PCa patient plasma; they proposed that these AR-originated circRNAs may serve as alternative circulatory biomarkers for AR expression and CRPC progression. Intriguingly, Luo et al. (2019) also identified an AR-derived circRNA circAR3, which is extremely upregulated in PCa patients' plasma, especially in advanced PCa patients. Additionally, they cannot be measured in patients' plasma after receiving radical prostatectomy; thus, they proposed that circAR3 is a promising biomarker in PCa. Meanwhile, some researchers reported that circZMIZ1 is highly expressed in PC patients' plasma and tissues compared with counterparts of BPH patients; circZMIZ1 drives the development of PCa by enhancing the expression levels of AR and AR splice variant 7 (Jiang et al., 2020). Chen et al. (2020) found that cRAPGEF5 is dramatically suppressed in RCC tissues compared with adjacent non-tumorous tissues, and among 245 cases of RCC, cRAPGEF5 reduction is closely associated with advanced clinical traits and can be an independent predictor for poor overall survival and recurrence-free survival in RCC. Moreover, an upregulated circular RNA in RCC tissues, circHIPK3, has been found to be positively associated with cancer stages, grades, and survival, and the receiver operating characteristic curve analysis indicated that circHIPK3 is a promising molecule marker in RCC (AUC = 0.95322, 95%CI: 0.9119–0.9945) (Han et al., 2020). Franz et al. (2019) proposed that circEGLN3 can act as a potential biomarker and discriminate malignant RCC from normal with an accuracy rate of 97%.

### Therapeutic Potential of Circular RNAs in Urological Cancers

As described earlier, the dysregulation of circRNAs may affect cancer cell proliferation, invasion, migration, angiogenesis, apoptosis, or drug resistance, thereby affecting the progression of a diversity of cancer. The advantage for targeting circRNA treatment lies in its potential of low off-target effects, whereas microRNAs and small interference RNAs have higher off-target effects owing to their short length and half-life. Recently, some researchers have artificially synthesized circRNAs (also named artificial sponges) to attenuate cardiac hypertrophy (Lavenniah et al., 2020) and suppress gastric carcinoma cell proliferation (Liu et al., 2018). These results suggest that it will be possible to treat urinary tumors by targeting circRNA. Chen Z. et al. (2020) illustrated that hsa_circ_001895 is upregulated in RCC tissues and cell lines; they utilized RNAi technology to silence the expression of has_circ_001895 and suppress tumor proliferation and metastasis *in vivo* and *in vitro*. Wang K. et al. (2017) found that AR can enhance ccRCC cell invasion and migration, whereas circHIAT1 can reverse this process, which may help us develop potential new treatments to inhibit ccRCC metastasis. Dong et al. (2019) research showed that circACVR2A is significantly downregulated in BC tissues and cell lines; overexpression of circACVR2A can dramatically restrain the proliferation and metastasis of BC. Meanwhile, upregulation of circRNA-MYLK was found to accelerate growth, angiogenesis, and migration of BC cell lines and xenografts in the study of Zhong et al. (2017); silencing the expression of circRNA-MYLK can inhibit the development of BC, which also indicated a potential therapeutic target for BC. Wu et al. (2019) preclinical study showed that circRNA17 could change the enzalutamide sensitivity and cell migration ability of CRPC cells by activating the miR-181c-5p/ARv7 signaling pathway, and this signaling pathway can be a better treatment method for enzalutamide-resistant CRPC. Shen et al. (2020) reported that circFOXO3 is significantly decreased in PCa patients, especially in advanced PCa patients, and disturbing the expression of circFoxo3 could accelerate PCa cells proliferation, metastasis, and chemotherapy resistance to docetaxel, which suggested that targeting circFoxo3 may be a viable strategy for PCa, especially for patients with docetaxel resistance. Given the crucial roles of circRNAs in the origination and development of urological cancers, it is reasonable to believe that circRNAs could be potential clinical therapeutic targets in the future.

## Conclusion and Perspectives

CircRNAs were initially regarded as nonfunctional “junks” generated by abnormal splicing events (Gao et al., 2016); with the recognition of novel RNA splicing events such as back-splicing and alternative splicing mechanisms, more and more functional circRNAs were identified, and plentiful researches elucidated that circRNAs are involved in diverse biological processes and disease progression, especially the genesis and development of human cancers. Urological cancers such as RCC, BC, and PCa were complicated diseases with high mortality and threatened human health. Therefore, a comprehensive review of the biogenesis, properties of circRNA, and its molecular mechanisms and clinical applications in urinary cancers may be helpful for the development of effective anticancer measures.

With the rapid development of high-throughput sequencing technology, plentiful circRNAs have been discovered, annotated, and functionally predicted (Fan et al., 2015). Owing to the special ring structure of circRNA and the high similarity of diverse transcript sequences of the same parental gene, the circRNA sequencing test usually has low accuracy and high false-positive rates; at this point, the process of circRNA validation is particularly important. However, many studies lack sufficient validations for circRNA; the main validation methods should include RT-PCR, sanger sequencing, Northern blot, RNA-FISH technologies, etc. (Kristensen et al., 2019). At the molecular mechanisms layer, so far, most studies of circRNAs have focused on miRNA sponges; the interactions between circRNAs and other novel mechanisms are still less. For example, do circRNAs regulate tumor progression by affecting novel cell programmed deaths such as ferroptosis, pyroptosis? The potential roles of circRNAs in the urinary tumor microenvironment remain unknown.

The ultimate goal of medical research is to improve survival; owing to the high stability and tissue-specific expression pattern of circRNAs, they have great potentials as molecular biomarkers and therapeutic targets. However, the samples of current researches are mainly derived from cancer tissues and cells; noninvasive samples such as urine, blood, and saliva were rarely applied to basic research. Additionally, for the purpose of treatment, the expression level of circRNAs needs to be controlled effectively; whether advanced technologies such as CRISPR-Cas-based editor systems can be applied to change the production of circRNAs without affecting their linear transcripts is also unclear. In brief, deep basic researches are needed to elucidate the underlying mechanisms, and reliable clinical studies are required to further validate the effectiveness of clinical applications of circRNAs. So that these findings can solve the actual clinical problems, thus improving the diagnosis and treatment in the future. CircRNAs are of great importance in the occurrence and development of urological cancers; we hope that with the development of biotechnology and basic research, more and more annotated circRNAs can be discovered and applied to clinical treatment.

## Abbreviations

AR, androgen receptor; AUC, area under the curve; BC, Bladder cancer; ceRNAs, competing endogenous RNAs; ciRNAs, circular intronic RNAs; CRPC, castration-resistant prostate cancer; ElciRNAs, exon-intron circRNAs; FN1, fibronectin 1; MREs, miRNA response elements; PCa, Prostate cancer; RCC, Renal cell carcinoma.
